# CPT2 as a Convergent Node Linking Age-Associated Neuronal H3K27me3 Remodeling to Nicotinamide Mononucleotide (NMN)-Induced Expression Rescue in Metabolic Tissues

**DOI:** 10.7759/cureus.112906

**Published:** 2026-07-18

**Authors:** Ngo Cheung

**Affiliations:** 1 Psychiatry, Cheung Ngo Medical Limited, Hong Kong, HKG

**Keywords:** cpt2, geroscience, h3k27me3, mitochondrial fatty acid oxidation, nad+ metabolism, neuronal epigenetic aging, nmn, polycomb repression, secondary data analysis, transcriptional rescue

## Abstract

Age-related decline in mitochondrial function and disruption of epigenetic regulation are two closely connected features of biological aging. In neurons, age-associated remodeling of repressive H3K27me3 chromatin may constrain genes needed for metabolic, synaptic, and stress-adaptive maintenance. In parallel, nicotinamide mononucleotide (NMN), an NAD+ precursor, has been reported to mitigate age-associated physiological and transcriptional changes in peripheral metabolic tissues. However, direct links between neuronal epigenetic aging programs and NMN-responsive transcriptional rescue remain unclear.

Here, we performed a secondary integrative analysis of two public datasets: GSE190102, focused on age-associated neuronal H3K27me3 targets mapped through an activity-by-contact-style region-gene framework, and GSE85718, a long-term NMN transcriptomic dataset from skeletal muscle, liver, and white adipose tissue in mice. The analysis identified 23 genes shared between 21,155 aging H3K27me3-associated targets and 35 robust NMN-rescue genes. Because the aging target set was extremely broad, gene-level overlap was not statistically persuasive, and pathway-level convergence was absent. Under repressive-mark direction logic, 14 of the 23 shared genes were concordant, meaning that the NMN expression effect opposed the expected consequence of age-associated H3K27me3 remodeling.

Objectives were to quantify overlap between neuronal age-associated H3K27me3 targets and robust NMN-responsive genes in peripheral metabolic tissues, classify shared genes by directional concordance under repressive chromatin logic, and identify high-priority mechanistic candidates. The analysis supports limited global convergence and nominates CPT2 as the leading convergent node for targeted validation.

CPT2 emerged as the leading candidate. It showed age-associated H3K27me3 gain, a large K27me3 log-fold change of +3.504, NMN-induced expression increase in old animals, a positive NMN interaction coefficient of +0.201, and membership in the mitochondrial fatty-acid oxidation pathway. Within the downstream shared-gene mitochondrial analysis, CPT2 was the only mitochondrial-core gene, with nominal enrichment only. These findings do not support a broad reversal of neuronal epigenetic aging by NMN. Instead, they identify CPT2 as a biologically coherent and experimentally tractable candidate linking age-related repressive chromatin remodeling to NMN-responsive mitochondrial metabolism.

## Introduction

Clinical/translational hook

Aging of the nervous system is not driven by one pathway. It reflects a gradual loss of cellular flexibility, including reduced mitochondrial reserve, impaired stress responses, altered chromatin regulation, and declining synaptic maintenance. These processes are relevant to cognitive aging, neurodegenerative vulnerability, and psychiatric states in which energy metabolism and stress adaptation are impaired. In clinical practice, this matters because many age-associated brain conditions are treated symptomatically, while the underlying metabolic and epigenetic constraints remain largely unaddressed.

Mitochondrial dysfunction has been placed among the major hallmarks of aging, alongside genomic instability, epigenetic alterations, altered nutrient sensing, and loss of proteostasis [1]. Studies of the aging human brain have also shown that genes involved in synaptic plasticity, vesicular transport, mitochondrial function, and neuronal survival are vulnerable during aging [2]. NAD+ biology has drawn attention because NAD+ is not only a redox metabolite but also a substrate for enzymes that regulate chromatin, stress responses, mitochondrial adaptation, and DNA repair [3-5]. These links make NAD+ metabolism attractive for translational work, but they also make it easy to overstate the implications. The present analysis was designed to stay narrow: it asks whether a small set of nicotinamide mononucleotide (NMN)-responsive transcriptional changes intersects with genes linked to neuronal H3K27me3 aging, and whether any candidate is strong enough to justify direct validation.

Epigenetic aging context

H3K27me3 is a repressive histone mark closely associated with Polycomb-mediated gene silencing. Polycomb group proteins are transcriptional repressors that help regulate gene expression during development and cell-state maintenance, and PRC2 is a major enzymatic complex involved in H3K27 methylation [6,7]. When H3K27me3 increases at regulatory regions, the expected direction is reduced transcriptional accessibility or reduced ability of a gene to respond to demand. In the context of aging neurons, this can be interpreted as a form of focal repression that may affect genes involved in neuronal identity, synaptic maintenance, and metabolic resilience. The related preprint by Cheung framed this process as focal Polycomb-mediated repression of neuronal identity and synaptic maintenance genes in aging neurons [8].

In this study, H3K27me3 gain was interpreted as increased repression, while H3K27me3 loss was interpreted as relative de-repression. This directionality is central. A simple gene overlap is not enough, because a gene can be shared between datasets while moving in a biologically contradictory direction. A more informative question is whether NMN changes expression in the direction that would oppose the inferred chromatin constraint.

NMN biology and rationale

NMN is an intermediate in NAD+ biosynthesis. Long-term NMN administration in mice has been reported to mitigate age-associated physiological decline and to prevent some age-associated transcriptional changes in metabolic tissues [9]. Broader reviews of NMN and nicotinamide riboside describe these molecules as NAD+ intermediates with potential biological and therapeutic relevance, while also emphasizing that translation to humans remains incomplete and context-dependent [10]. In a human trial, NMN increased muscle insulin sensitivity in prediabetic women, but such findings should not be generalized to all aging outcomes or to brain aging without additional evidence [11].

Mechanistically, NAD+ can influence sirtuin activity. AMPK has been shown to increase cellular NAD+ and thereby enhance SIRT1 activity, leading to deacetylation of downstream targets such as PGC-1alpha and FOXO transcription factors [12]. SIRT1 can interact with PGC-1alpha, a regulator of mitochondrial biogenesis and oxidative metabolism [13,14]. SIRT3 is localized to mitochondria and regulates mitochondrial protein acetylation, including metabolic pathways important for energy homeostasis [15,16]. These mechanisms provide a plausible bridge between NAD+ repletion, mitochondrial function, and chromatin-linked metabolic regulation, but they do not prove that NMN directly erases H3K27me3 at specific genes.

The related preprint by Cheung identified mechanistic leads from NMN-associated transcriptional drift, including RAB11A-linked trafficking and CPT2-linked fatty-acid oxidation [17]. The current manuscript builds from that logic but uses a stricter interpretation: the strongest claim is not that NMN reverses epigenetic aging, but that some NMN-responsive genes may overlap with chromatin-constrained aging targets in a way that points to testable candidates.

Study rationale and gap

The unresolved question is whether genes linked to neuronal age-associated H3K27me3 remodeling overlap with genes whose age-associated expression trajectories are reversed by NMN in metabolic tissues. This is not a direct test of NMN action in neurons. The comparison crosses tissue, assay type, age range, and molecular layer. It is therefore best understood as candidate discovery.

This indirect design still has value. If a gene is linked to increased repressive chromatin in aging neurons and is also transcriptionally rescued by NMN in old metabolic tissues, that gene may sit near a shared aging axis. The strongest candidates would be genes with directional coherence, plausible biology, and feasible experimental readouts. The present analysis identified CPT2 as the clearest example.

Objectives

The four objectives were: (1) To quantify overlap between neuronal H3K27me3 aging targets and transcriptomic rescue genes from non-neuronal metabolic tissues, specifically skeletal muscle, liver, and white adipose tissue; (2) To classify shared genes by directional concordance under repressive H3K27me3 logic; (3) To identify the strongest mechanistic candidate, with particular attention to CPT2; (4) To discuss translational implications and a validation roadmap while preserving the analytical limitations and candidate-discovery scope of the study.

## Materials and methods

Data sources

**Figure 1:**
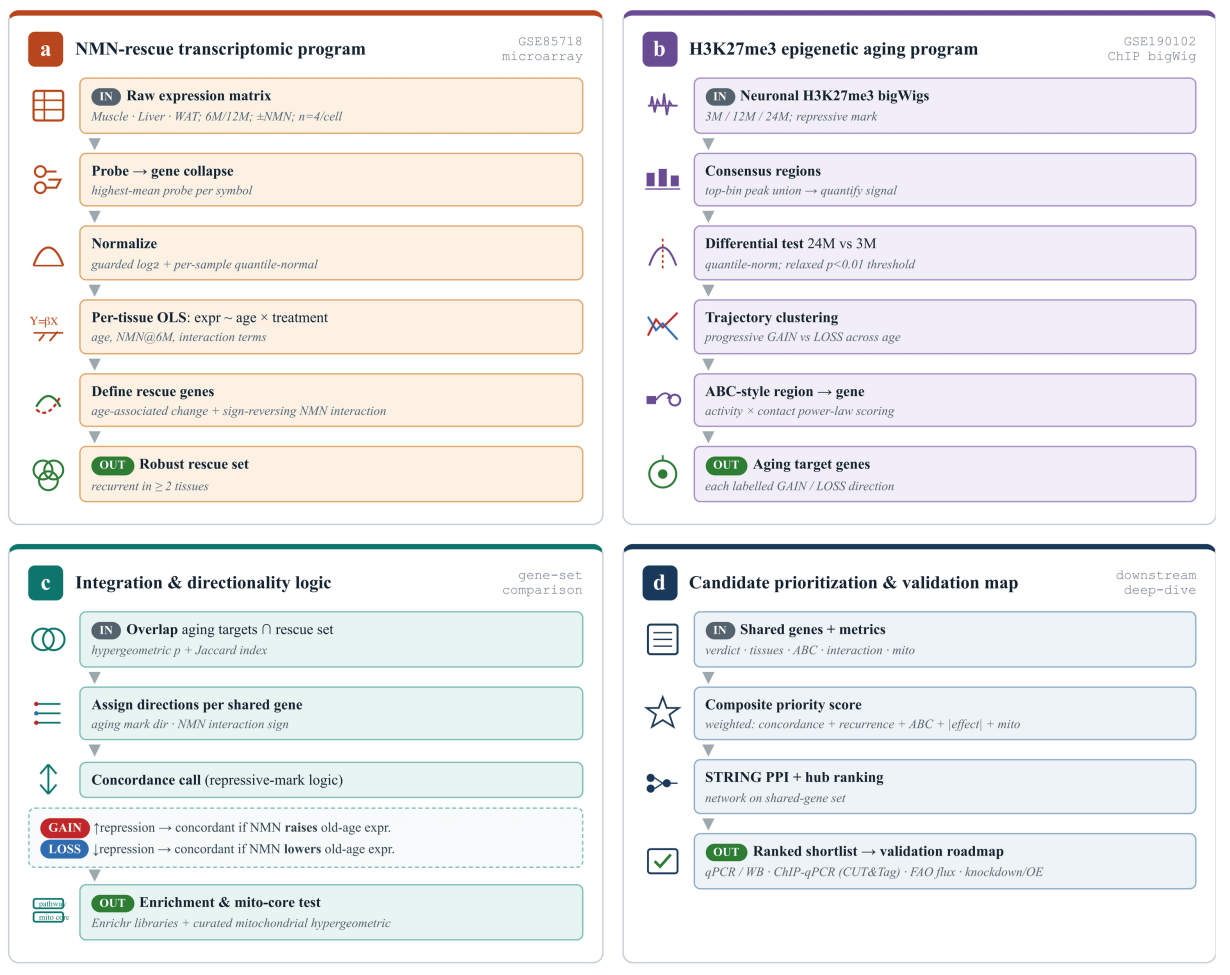
Integrative secondary-analysis pipeline linking neuronal H3K27me3 aging targets to NMN-responsive transcription. (a) The nicotinamide mononucleotide transcriptomic dataset Gene Expression Omnibus Series GSE85718 was processed from the raw expression matrix through probe-to-gene collapse, guarded logarithm base 2 and quantile normalization, and per-tissue ordinary-least-squares modeling of expression as a function of age, treatment, and their interaction; rescue genes were defined by an age-associated change accompanied by a sign-reversing nicotinamide mononucleotide interaction and retained if recurrent in at least two tissues. (b) The neuronal histone H3 lysine 27 trimethylation dataset Gene Expression Omnibus Series GSE190102 was processed from bigWig signal tracks into consensus regions, differentially tested for the 24-month-versus-3-month contrast, clustered by age trajectory into progressive-gain and progressive-loss sets, and linked to protein-coding genes using an activity-by-contact-style region-gene framework, yielding directionally labeled aging target genes. (c) The two gene programs were intersected; each shared gene was assigned an aging-mark direction and a nicotinamide mononucleotide interaction sign, and a concordance call was made under repressive-mark logic: histone H3 lysine 27 trimethylation gain is expected to be opposed by a nicotinamide mononucleotide-induced expression increase, and loss by a nicotinamide mononucleotide-induced decrease. This was followed by pathway enrichment and a curated mitochondrial-core test. (d) Shared genes were scored by a composite priority combining directional concordance, cross-tissue recurrence, region-gene support, interaction magnitude, and mitochondrial membership, refined with a protein-protein interaction network, and mapped onto a tiered experimental validation roadmap. This figure depicts the analytical workflow only; no results are shown. NMN, nicotinamide mononucleotide. Credits to Ngo Cheung; created using PowerPoint (Microsoft Corporation, Redmond, WA, USA); no artificial intelligence used.

This study used secondary analysis of public datasets and pipeline outputs derived from them (Figure 1). The aging epigenetic program was based on GSE190102, analyzed as neuronal H3K27me3 signal across age groups, with 3-month and 24-month mice serving as the main young-versus-old contrast. The NMN-rescue program was based on GSE85718, corresponding to long-term NMN administration in C57BL/6N mice. The NMN dataset included skeletal muscle, liver, and white adipose tissue, with 6-month and 12-month age groups and control or NMN treatment. Each tissue, age, and treatment cell had four samples. The original long-term NMN mouse study used oral NMN and reported mitigation of age-associated physiological decline and tissue-dependent transcriptional effects [9]. A complete tabulated master overview is provided in Appendices 1-15.

Aging epigenetic targets

For the aging analysis, H3K27me3 bigWig files were processed to define consensus regions and quantify age-associated signal. The detected histone mark was K27me3, treated as a repressive mark rather than an active enhancer mark. Differential selection used a relaxed threshold of p < 0.01 when stricter criteria produced too few regions. The pipeline identified 9,752 age-associated regions, of which 6,402 progressive gain or loss regions were carried into region-gene assignment. The activity-by-contact-style framework was used to link regions to protein-coding genes, producing 427,099 region-gene pairs and 21,155 target genes. The numerical ABC-style score cutoff used to retain these region-gene pairs will be reported in the deposited parameter file; the aggregate tabulated results establish the retained-pair and target-gene counts but do not permit that cutoff to be inferred. Because H3K27me3 is repressive, “activity” in this context should be read as mark intensity at Polycomb-associated or heterochromatin-like loci, not as enhancer activation. The activity-by-contact model itself is a general enhancer-promoter prediction framework based on regulatory activity and contact frequency [18].

The complete aging parameter summary and mitochondrial-core list are provided in Appendices 6 and 7.

NMN-rescue genes

For GSE85718, 25,697 probes were collapsed to 18,120 genes. Values were already compressed, so log2 transformation was skipped, and quantile normalization was applied. Within each tissue, the model was expressed as a function of age, treatment, and age-by-treatment interaction. No genes reached FDR < 0.05 for the age effect, the NMN effect at 6 months, or the interaction term in any tissue. Because of this, the rescue definition used relaxed nominal criteria: a gene had to show an age-associated expression change in control animals and a sign-reversing NMN interaction. Genes were considered robust if they appeared in at least two tissues. This produced 421 rescue genes in skeletal muscle, 355 in liver, 397 in white adipose tissue, and 35 robust cross-tissue rescue genes. No gene was rescued in all three tissues.

The exact nominal p-value thresholds used for the relaxed rescue calls will be specified in the deposited parameter table and analysis scripts. The detailed tissue-level and cross-tissue NMN outputs are reported in Appendices 3-5.

Overlap and directionality analysis

The primary overlap compared the 21,155 aging H3K27me3-associated target genes with the 35 robust NMN-rescue genes. Directionality was interpreted according to repressive H3K27me3 logic. A gene with H3K27me3 gain with age was expected to be more repressed; therefore, an NMN-associated expression increase in old animals was classified as concordant. A gene with H3K27me3 loss with age was expected to be less repressed; therefore, an NMN-associated expression decrease in old animals was classified as concordant. Other combinations were classified as discordant.

The overlap metrics are also tabulated in Appendix 8.

Mitochondrial analysis

A curated mitochondrial and energy gene core was used to test whether shared genes were enriched for mitochondrial biology. This core included oxidative phosphorylation genes, tricarboxylic acid cycle genes, mitochondrial dynamics genes, fatty acid oxidation genes, NAD-related mitochondrial regulators, and related energy metabolism genes. Hypergeometric testing was applied, but results were interpreted cautiously because the target universe and gene-selection procedure were not ideal for formal enrichment claims.

Enrichment

Gene-set enrichment was performed using Enrichr libraries, including Gene Ontology Biological Process, KEGG Mouse, Reactome, MSigDB Hallmark, and MGI Mammalian Phenotype terms. Enrichr was applied to the full aging target set, the robust NMN set, the shared-gene set, and the concordant and discordant subsets. Enrichr is a widely used web-based gene-list enrichment tool, but the lack of a custom background universe is an important limitation in this setting [19,20].

Statistical considerations

Several statistical constraints were present from the start. The aging target list contained 21,155 genes, nearly the full eligible protein-coding space in the analysis. This makes gene-level overlap difficult to interpret, because almost any small gene list will overlap with such a broad set. In addition, the stated hypergeometric universe in the master pipeline was 20,000, which was smaller than the aging target count. That made the reported hypergeometric p-value invalid and produced NaN. The correct interpretation is not that the overlap is formally significant or formally non-significant under that test, but that it is not interpretable as compelling evidence of convergence under the current universe definition. A better universe would be the intersection of genes measurable in GSE85718 and genes eligible for GSE190102 ABC-style mapping.

The attempted gene universe, the resulting NaN value, and the related statistical constraints are summarized in Appendix 2. The corresponding overlap metrics are provided in Appendix 8.

Data and code availability

The public source datasets are available through GEO under GSE190102 and GSE85718. The computational workflow and analysis parameters are available in the public GitHub repository, which contains the 088EvA.ipynb analysis notebook and accompanying documentation [21].

The analysis parameters and numerical thresholds are specified within the code provided in the public GitHub repository. These include the H3K27me3 selection criterion of p < 0.01, the FDR criterion of FDR < 0.05, the relaxed nominal thresholds used for rescue calls, the mitochondrial-core test parameters, and the ABC-style score cutoff used to retain 427,099 region-gene pairs and generate 21,155 aging target genes. The numbered appendices, specifically Appendices 1-15, report the retained-pair and target-gene counts but do not state the numerical ABC cutoff; that value is specified in the original analysis code and should not be inferred from the downstream CPT2 score of 0.0698.

Supporting machine-readable tables, datasets, and analysis scripts are available upon request. These include scripts for data processing, normalization, statistical analyses, region-gene integration, enrichment analyses, and hypergeometric testing, as well as the complete mitochondrial-core gene list, the 35 robust NMN-rescue genes, the 23 shared genes, intermediate output files required to reproduce the reported Jaccard index, enrichment results, and invalid hypergeometric result, attempted universe of 20,000 genes and the 21,155-gene aging target count, including the reason the hypergeometric calculation returned NaN.

## Results

The key input datasets, processing outputs, and interpretive constraints are summarized in Table 1. Detailed master-analysis outputs are also provided in Appendices 1 and 2.

**Table 1:** Master analysis overview and principal constraints. NMN, nicotinamide mononucleotide; H3K27me3, histone H3 lysine 27 trimethylation; K27me3, histone H3 lysine 27 trimethylation; GSE, Gene Expression Omnibus series; ABC, activity-by-contact; CPT2, carnitine palmitoyltransferase 2; FDR, false discovery rate.

Component	Result	Interpretation
Aging dataset	GSE190102	Neuronal H3K27me3 aging signal; 24M versus 3M contrast; ABC-style region–gene mapping
NMN dataset	GSE85718	Long-term oral NMN, 300 mg/kg/day; C57BL/6N mice; skeletal muscle, liver, and white adipose tissue; 6M and 12M
Aging stage	Completed	Aging H3K27me3 target construction completed
NMN stage	Completed	NMN-rescue transcriptomic analysis completed
Main interpretation	Limited global convergence	No shared significant pathway terms; 23 shared genes identified
Top validation candidate	CPT2	Concordant, mitochondrial, K27me3 gain, NMN-induced expression increase in old animals
Broad aging target set	21,155 genes	Weakens binary overlap interpretation
Relaxed NMN-rescue criteria	Used because no FDR-significant tissue-specific effects were detected	Supports candidate discovery rather than formal rescue claims
Primary conclusion	Candidate discovery	Supports CPT2-focused validation rather than broad claims that NMN reverses neuronal epigenetic aging

Gene-level overlap and limitations

Objective 1

Gene-level overlap analysis compared the neuronal H3K27me3 target set with the transcriptomic rescue set from non-neuronal metabolic tissues. The aging H3K27me3 analysis produced 21,155 target genes. The robust NMN-rescue analysis produced 35 genes present in at least two tissues. The intersection contained 23 genes: ACD, APBA2, BCHE, CMA2, CPT2, DUSP11, FMO2, GALR1, HS3ST6, MAP2K2, MYO15, MYOM2, OLFR508, PKNOX1, PSME3, RAB11A, RANGAP1, SLC13A5, SPG21, SYNGR2, TRP53INP2, WAP, and ZFAND2B.

The NMN-rescue transcriptomic program and cross-tissue filtering are summarized in Table 2, and the aging H3K27me3 program is summarized in Table 3. The corresponding detailed appendix outputs are provided in Appendices 3-7.

**Table 2:** NMN-rescue transcriptomic program and robust cross-tissue filtering. NMN, nicotinamide mononucleotide; FDR, false discovery rate.

Tissue or category	Age/treatment design	n	Differential genes at FDR < 0.05	Relaxed NMN-rescue result
Liver	6M control, 6M NMN, 12M control, 12M NMN	4 per cell	Age effect: 0; NMN at 6M: 0; age × treatment interaction: 0	355
Skeletal muscle	6M control, 6M NMN, 12M control, 12M NMN	4 per cell	Age effect: 0; NMN at 6M: 0; age × treatment interaction: 0	421
White adipose tissue	6M control, 6M NMN, 12M control, 12M NMN	4 per cell	Age effect: 0; NMN at 6M: 0; age × treatment interaction: 0	397
Probe-to-gene processing	25,697 probes collapsed to genes	—	—	18,120 genes
Robust rescue genes	Present in at least two tissues	—	—	35
Rescued in all three tissues	Present in liver, skeletal muscle, and white adipose tissue	—	—	0
NMN mitochondrial annotation	CPT2, CS, NDUFV1, POLG, SUCLA2, TFB2M	—	—	6 genes

**Table 3:** Aging H3K27me3 epigenetic program. H3K27me3, histone H3 lysine 27 trimethylation; K27me3, histone H3 lysine 27 trimethylation; ABC, activity-by-contact; GENCODE, GENCODE gene annotation database.

Parameter	Value	Note
Histone mark	K27me3 / H3K27me3	Repressive mark; not treated as active enhancer signal
Samples per age	3M: 5; 12M: 5; 24M: 5	Age groups parsed from 15 K27me3 bigWig files
Consensus regions	366,721	Peak-union regions used for quantification
Differential mode	Relaxed p < 0.01	24M versus 3M contrast
Age-associated regions	9,752	Regions selected for age-associated K27me3 change
Progressive loss clusters	1, 4	K27me3 signal progressively decreased with age
Progressive gain clusters	0, 3	K27me3 signal progressively increased with age
Regions carried to ABC	6,402	Progressive gain or loss regions used for region–gene scoring
Protein-coding genes	21,674	GENCODE vM25 protein-coding genes considered
ABC region–gene pairs	427,099	Predictions above the ABC-style score threshold
Aging K27me3 target genes	21,155	GAIN = 9,178; LOSS = 12,470
Mitochondrial-core genes in aging target set	66	Included CPT2, CS, NDUFV1, POLG, SUCLA2, TFB2M, and other energy-core genes

At first glance, 23 of 35 robust NMN genes overlapping with the aging set appear large. However, the aging target set was so broad that the overlap was not statistically significant. The Jaccard index was only 0.0011. The hypergeometric p-value was reported as NaN because the predefined universe was 20,000, smaller than the 21,155 aging target genes. This invalidates the formal overlap test. The more accurate statement is that the exact gene overlap is hypothesis-generating but not strong evidence for shared biology. These overlap metrics are tabulated in Appendix 8.

Pathway-level comparison was even more conservative. The aging target set produced 489 significant Enrichr terms at adjusted p < 0.05, while the robust NMN set produced zero significant terms. Consequently, there were zero shared significant pathway terms. The top aging terms included ABC transporters, ion channel transport, adrenergic signaling in cardiomyocytes, ion transport by P-type ATPases, transport of small molecules, aldosterone synthesis and secretion, RHOA GTPase cycle, insulin receptor recycling, cGMP-PKG signaling, and O-glycosylation-related terms. These results support the view that the neuronal H3K27me3 aging program is broad and pathway-rich, while the robust NMN set is small and underpowered for enrichment. The full pathway comparison and functional theme counts are provided in Appendices 10-12.

Directional concordance

Objective 2

Directional concordance analysis classified 14 of the 23 shared genes as concordant and 9 as discordant under repressive H3K27me3 logic.

The most informative part of the comparison was not the raw overlap but the direction of effect. Because H3K27me3 is a repressive mark, H3K27me3 gain with age suggests increased repression, while H3K27me3 loss suggests reduced repression. A rescue-like NMN effect should oppose the predicted expression consequence of that chromatin change.

Using this logic, 14 of the 23 shared genes were concordant, and 9 were discordant. The concordant genes were ACD, APBA2, BCHE, CPT2, DUSP11, FMO2, GALR1, HS3ST6, MAP2K2, MYOM2, PKNOX1, SPG21, SYNGR2, and ZFAND2B. The discordant genes were CMA2, MYO15, OLFR508, PSME3, RAB11A, RANGAP1, SLC13A5, TRP53INP2, and WAP.

The shared genes and direction calls are shown in Table 4. The complete shared-gene direction table is also provided in Appendix 9.

**Table 4:** Shared genes with direction and concordance classification. K27me3, histone H3 lysine 27 trimethylation; NMN, nicotinamide mononucleotide.

Gene	Aging K27me3 direction	NMN effect in old animals	NMN sign	Verdict
ACD	LOSS	NMN lowers old	-1.0	Concordant
APBA2	GAIN	NMN raises old	+1.0	Concordant
BCHE	GAIN	NMN raises old	+1.0	Concordant
CMA2	LOSS	NMN raises old	+1.0	Discordant
CPT2	GAIN	NMN raises old	+1.0	Concordant
DUSP11	LOSS	NMN lowers old	-1.0	Concordant
FMO2	LOSS	NMN lowers old	-1.0	Concordant
GALR1	GAIN	NMN raises old	+1.0	Concordant
HS3ST6	GAIN	NMN raises old	+1.0	Concordant
MAP2K2	LOSS	NMN lowers old	-1.0	Concordant
MYO15	LOSS	NMN raises old	+1.0	Discordant
MYOM2	LOSS	NMN lowers old	-1.0	Concordant
OLFR508	GAIN	NMN lowers old	-1.0	Discordant
PKNOX1	LOSS	NMN lowers old	-1.0	Concordant
PSME3	LOSS	NMN raises old	+1.0	Discordant
RAB11A	LOSS	NMN raises old	+1.0	Discordant
RANGAP1	LOSS	NMN raises old	+1.0	Discordant
SLC13A5	LOSS	NMN raises old	+1.0	Discordant
SPG21	LOSS	NMN lowers old	-1.0	Concordant
SYNGR2	LOSS	NMN lowers old	-1.0	Concordant
TRP53INP2	LOSS	NMN raises old	+1.0	Discordant
WAP	GAIN	NMN lowers old	-1.0	Discordant
ZFAND2B	LOSS	NMN lowers old	-1.0	Concordant

This classification created a more useful validation framework than overlap alone. The concordant genes are candidates where the NMN expression effect is consistent with the reversal of the inferred H3K27me3-associated expression constraint. The discordant genes should not be dismissed because they may reflect tissue-specific regulation, indirect NMN effects, compensatory responses, or uncertainty in distal region-gene mapping. Still, the concordant set is more directly aligned with the rescue model.

CPT2 as the leading candidate

Objective 3

Candidate prioritization identified CPT2 as the leading validation candidate.

CPT2 was the clearest candidate in the analysis. It appeared in the shared gene set, was classified as concordant, belonged to the curated mitochondrial core, and ranked first in the downstream validation priority score. Its aging-side signal was H3K27me3 gain, with a K27me3 log-fold change of +3.504. Under repressive-mark logic, this suggests increased age-associated chromatin constraint at the CPT2 locus or linked regulatory region. Its NMN-side signal was a positive interaction coefficient of +0.201, meaning that NMN increased CPT2 expression in old animals relative to the expected age effect. CPT2 was robust across two metabolic tissues, had a mean age-control coefficient of -0.141, and had an ABC score of 0.0698 in the downstream target table.

CPT2-focused metrics and validation priorities are summarized in Table 5. The downstream shortlist and CPT2-focused metrics are provided in Appendices 13 and 14.

**Table 5:** CPT2-focused result summary and validation roadmap. CPT2, carnitine palmitoyltransferase 2; NMN, nicotinamide mononucleotide; K27me3, histone H3 lysine 27 trimethylation; LFC, log-fold change; ABC, activity-by-contact; qPCR, quantitative polymerase chain reaction; ChIP-qPCR, chromatin immunoprecipitation–quantitative polymerase chain reaction; CUT&Tag, cleavage under targets and tagmentation.

Metric or validation item	CPT2 value or recommendation	Interpretation
Shared gene status	Shared	Present in both the aging K27me3 target set and the robust NMN-rescue set
Aging K27me3 direction	GAIN	Consistent with increased age-associated repressive chromatin signal
K27me3 LFC, 24M versus 3M	+3.504	Large positive age-associated K27me3 change
NMN effect	NMN raises old	Positive expression interaction in old animals, consistent with rescue under repressive-mark logic
Mean NMN interaction coefficient	+0.201	Expression increased with NMN in old animals
Mean age-control coefficient	-0.141	Age effect in control animals was negative on average
Number of NMN tissues	2	Met robust criterion of presence in at least two tissues
ABC score	0.0698	Region–gene support from the downstream aging target table
Mitochondrial-core status	Yes	Only mitochondrial-core gene in the strict 23-gene shared downstream set
Downstream priority score	7.91	Highest-ranked validation candidate
Priority 1 validation	qPCR and Western blot for CPT2	Confirms whether CPT2 expression is reproducibly age-sensitive and NMN-responsive
Priority 2 validation	H3K27me3 ChIP-qPCR or CUT&Tag-qPCR at implicated CPT2 regulatory regions	Tests whether age-associated K27me3 gain is reproducible and NMN-sensitive
Priority 3 validation	Fatty-acid oxidation assays using Seahorse flux or labeled palmitate, with and without NMN	Determines whether CPT2-linked expression changes correspond to altered fatty-acid oxidation capacity
Priority 4 validation	CPT2 knockdown or overexpression combined with NMN treatment	Tests whether CPT2 is required for, or sufficient to mimic, part of the NMN-associated metabolic response
Priority 5 validation	Concordant-gene qPCR panel	Assesses whether a small K27me3-coherent NMN-response signature can be replicated
Priority 6 validation	Recompute overlap using a valid universe and rank-based target selection	Improves formal interpretation of overlap, expected overlap, odds ratio, and enrichment

The biological fit is strong. CPT2 encodes carnitine palmitoyltransferase 2, a key enzyme of the mitochondrial carnitine shuttle. The carnitine shuttle is required for long-chain fatty acids to enter mitochondrial beta-oxidation, and CPT2 functions at the inner mitochondrial membrane side of this system [22-24]. Defects in this pathway impair fatty-acid oxidation and can cause clinically meaningful metabolic disease. In the present analysis, CPT2 was not simply a shared gene; it was the only gene in the downstream 23-gene shared set that belonged to the curated mitochondrial core. The mitochondrial enrichment among the shared genes was nominal, with hypergeometric p = 0.0732, and therefore should be described as suggestive rather than significant.

There is a distinction between the broader mitochondrial analysis and the downstream shared-gene result. In the master pipeline, six mitochondrial-core genes overlapped between the full aging mitochondrial set and the broader NMN robust/union mitochondrial annotation: CPT2, CS, NDUFV1, POLG, SUCLA2, and TFB2M. In the stricter downstream shared robust set of 23 genes, only CPT2 remained as a mitochondrial-core candidate. This is why CPT2 is the cleanest mechanistic bridge, not because it proves a general mitochondrial overlap, but because it is the one mitochondrial gene that satisfies the strongest combined filters.

Broader concordant and discordant patterns

The downstream priority score weighted concordance, tissue recurrence, ABC rank, absolute NMN interaction strength, and mitochondrial membership. CPT2 ranked first with a score of 7.91. Other high-priority concordant genes included ACD, ZFAND2B, FMO2, SYNGR2, DUSP11, APBA2, MAP2K2, SPG21, HS3ST6, PKNOX1, MYOM2, GALR1, and BCHE. ACD and ZFAND2B ranked highly because of concordance and favorable ABC-related evidence. FMO2 is interesting because flavin-containing monooxygenases have been linked in some contexts to detoxification and stress resistance, although the effect size here was small. SYNGR2 and APBA2 point toward membrane- or vesicle-associated biology, which may be relevant to neuronal maintenance but requires tissue-specific validation.

The discordant set showed the strongest enrichment signal. Concordant genes had zero significant enrichment terms, while the 9 discordant genes produced 38 significant GO terms. These terms were mostly related to succinate transport, response to lithium ion, neurotransmitter receptor transport from endosome to postsynaptic membrane or plasma membrane, plasma membrane-to-endosome transport, regulation of endosome size, astral microtubule organization, C4-dicarboxylate transport, and negative regulation of nucleocytoplasmic transport. Because the discordant group contained only 9 genes, this enrichment may be unstable. Still, it suggests that NMN-related effects may include adaptive trafficking and transport responses that do not map neatly onto direct reversal of neuronal H3K27me3 repression. Representative discordant enrichment terms are listed in Appendix 15.

This pattern suggests that NMN may not act as a simple chromatin “reversal” agent. It may have at least two arms: a candidate direct or directionally coherent rescue arm, represented by genes such as CPT2, and an adaptive remodeling arm, represented by transport and trafficking terms in the discordant set. This distinction matters for translational work. A therapy that improves aging physiology may still produce tissue-specific or compensatory expression patterns that do not mirror the original aging mark.

Methodological constraints

The results must be read with caution. The aging target set was extremely broad. The NMN-rescue genes were defined using relaxed nominal criteria because no genes reached genome-wide FDR significance in the tissue-specific models. The two datasets differ in tissue, age range, assay modality, and molecular layer. The analysis therefore supports candidate discovery, not causal inference, and not broad claims about NMN reversing neuronal epigenetic aging.

## Discussion

Mechanistic synthesis for CPT2

**Figure 2:**
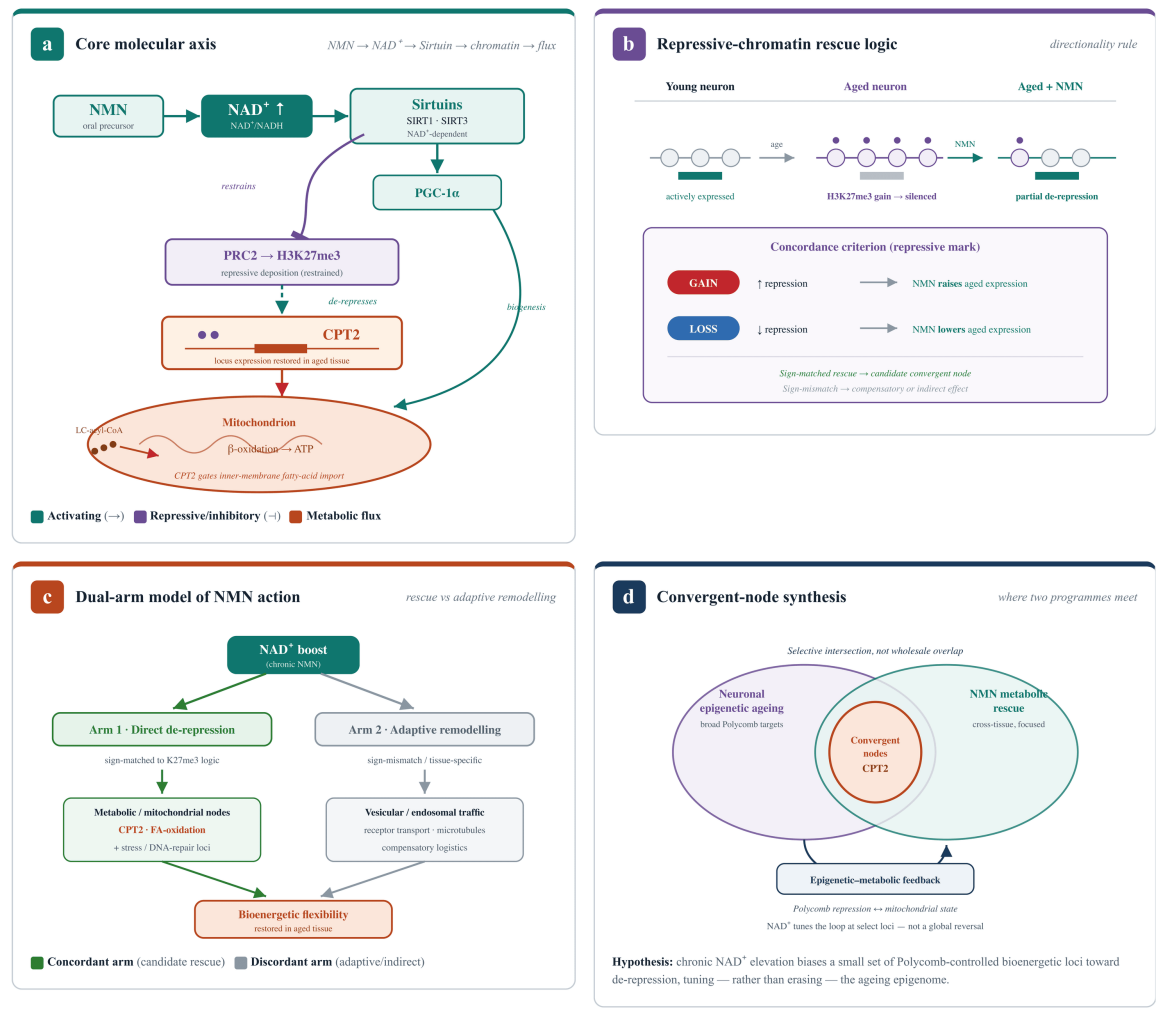
Proposed NAD⁺-Sirtuin-Polycomb axis coupling epigenetic ageing to mitochondrial fatty-acid oxidation. (a) Proposed core axis: Oral nicotinamide mononucleotide (NMN) may increase cellular NAD+ and the NAD+/NADH ratio, activating NAD^+^-dependent sirtuins such as SIRT1 and SIRT3. This is hypothesized to restrain Polycomb repressive complex 2-mediated H3K27me3 deposition, enhance PGC-1α-driven mitochondrial biogenesis, relieve repression at the CPT2 locus, and support carnitine-dependent import of long-chain acyl-CoA for mitochondrial β-oxidation. (b) Directionality rule for coherent rescue: Age-related H3K27me3 gain, which increases silencing, is considered opposed by an NMN-induced increase in aged-tissue expression; H3K27me3 loss is opposed by an NMN-induced decrease. Sign-matched genes are candidate convergent nodes, whereas sign-mismatched genes may reflect compensation or indirect effects. (c) Dual-arm model: NAD+ elevation may act through a concordant arm that de-represses metabolic and mitochondrial loci, led by CPT2 and fatty-acid oxidation, and a discordant arm that remodels vesicular and endosomal trafficking. Both may converge on restored bioenergetic flexibility. (d) Synthesis: The broad neuronal Polycomb-aging program and the focused NMN metabolic-rescue program may intersect at a limited set of convergent nodes within an epigenetic-metabolic feedback loop that NAD+ selectively tunes rather than globally reverses. All molecular relationships, pathways, and mechanisms shown are hypothesized predictive models based on literature integration and secondary analysis. This study provides no direct experimental evidence that NMN alters H3K27me3 deposition at specific loci or any causal chromatin-level data. Proposed links - including NAD^+^–Sirtuin-Polycomb and CPT2-regulatory relationships - remain unvalidated and require targeted experimental testing. Credits to Ngo Cheung; created using PowerPoint (Microsoft Corporation, Redmond, WA, USA); no artificial intelligence used.

The central findingo is narrow but biologically coherent: CPT2 sits at the intersection of age-associated neuronal H3K27me3 gain and NMN-associated expression rescue in old metabolic tissues. The simplest model is that aging increases repressive Polycomb-linked chromatin at or near CPT2-associated regulatory regions, which may constrain CPT2 expression or reduce its responsiveness to metabolic demand. NMN, by increasing NAD+ availability and engaging sirtuin-linked metabolic programs, may partially restore expression in old tissue. This model is plausible but not yet proven (Figure 2).

Polycomb biology supports the epigenetic side of the model. PRC-associated systems help maintain transcriptional repression, and H3K27me3 is a major feature of Polycomb-linked chromatin states [6,7]. The neuronal aging preprint by Cheung extends this logic to aging neurons, proposing focal Polycomb-mediated repression of neuronal identity and synaptic maintenance genes [8]. In the present analysis, CPT2 showed H3K27me3 gain with age and a large K27me3 log-fold change of +3.504. That makes it one of the clearest loci where a repressive aging mark and an NMN-rescue expression signal point in the same direction.

The metabolic side of the model is equally direct. CPT2 is part of the mitochondrial fatty acid oxidation system. Long-chain fatty acids require the carnitine shuttle for mitochondrial entry and oxidation, and CPT2 is needed to regenerate long-chain acyl-CoA inside the mitochondrial compartment for beta-oxidation [22-24]. If CPT2 expression or regulation is constrained during aging, the cell may lose some flexibility in using fatty acids as energy substrates. This does not mean that CPT2 alone determines mitochondrial aging, but it does make CPT2 a practical readout of fatty-acid oxidative capacity.

The NMN side of the model likely involves NAD+ and sirtuins rather than direct action on H3K27me3. AMPK can increase NAD+ and enhance SIRT1 activity, leading to deacetylation of PGC-1alpha and FOXO targets [12]. SIRT1 interacts with PGC-1alpha, while SIRT3 is mitochondrial and influences acetylation of metabolic proteins [13,15,16]. NAD+ repletion has also been linked to mitochondrial and stress-response programs in aging models [25,26]. Thus, NMN may improve the metabolic context in which CPT2 is regulated. It may also indirectly affect chromatin modifier recruitment through redox state, acetylation state, mitochondrial stress signaling, or transcription factor activity. However, this analysis does not show that NMN removes H3K27me3 from CPT2. That remains a testable hypothesis.

A reasonable working model is therefore an epigenetic-metabolic feedback loop. Aging increases repressive chromatin pressure at CPT2-linked regions. This may reduce fatty-acid oxidation capacity or narrow metabolic flexibility. NMN raises NAD+ availability, supports sirtuin-linked mitochondrial programs, and increases CPT2 expression in old tissue. If validated, CPT2 would represent a specific node where chromatin aging and metabolic rescue meet.

Why global convergence is limited

The analysis did not show broad convergence between neuronal H3K27me3 aging and NMN-induced transcriptional rescue. This is not surprising. The aging side was neuronal and chromatin-based, using H3K27me3 in a 24-month versus 3-month contrast. The NMN side was transcriptomic, measured in skeletal muscle, liver, and white adipose tissue, using 12-month versus 6-month animals and an age-by-treatment interaction model. These are different biological layers and different aging windows.

The gene-selection asymmetry was also substantial. The aging target set contained 21,155 genes, almost the entire eligible protein-coding space. This makes overlap almost unavoidable and weakens standard enrichment testing. The NMN robust set contained only 35 genes and was based on relaxed nominal p-value criteria, although the requirement for recurrence in at least two tissues added some protection against tissue-specific noise. Together, these features explain why exact overlap is not a strong finding and why pathway convergence was absent.

Prior NMN omics literature also warrants a balanced reading. The original long-term mouse study reported physiological benefit but described tissue-dependent transcriptional effects [9]. Reviews of NMN and nicotinamide riboside emphasize that responses vary with tissue, age, dose, and experimental context, and that translation to humans remains incomplete [10]. The human study in prediabetic women demonstrated a defined metabolic benefit, not a universal transcriptomic or brain-aging rescue [11]. Published NMN-related work, therefore, does not establish a uniformly consistent rescue signature. The present findings are more consistent with a tissue-specific and context-dependent framework than with a model of universal transcriptional or chromatin normalization.

The best framing is therefore not “NMN reverses neuronal epigenetic aging.” The data do not support that. A more defensible interpretation is that a small number of genes, especially CPT2, meet criteria for directional and biological plausibility across two aging-related programs. These genes are candidates for targeted validation.

Discordant signal - compensatory versus direct rescue

The discordant genes deserve attention because they may point to biology that is not captured by the simple rescue model. Discordance can occur when neuronal chromatin and metabolic-tissue expression are regulated differently. It can also occur when NMN affects a pathway indirectly, as part of a compensatory or homeostatic response. For example, improved mitochondrial function or altered NAD+/NADH balance could change endosomal trafficking, receptor recycling, or protein turnover without directly opposing a neuronal H3K27me3 mark.

The discordant group included SLC13A5, CMA2, WAP, TRP53INP2, PSME3, OLFR508, MYO15, RAB11A, and RANGAP1. These genes produced 38 significant GO terms, many related to endosome-to-plasma membrane transport, neurotransmitter receptor trafficking, microtubule organization, succinate transport, and C4-dicarboxylate transport. This was the most statistically visible enrichment in the downstream analysis, but the gene count was small. It should be treated as a lead, not as a stable pathway claim.

This pattern suggests that NMN may not act as a simple chromatin “reversal” agent. It may have at least two arms: a candidate direct or directionally coherent rescue arm, represented by genes such as CPT2, and an adaptive remodeling arm, represented by transport and trafficking terms in the discordant set. This distinction matters for translational work. A therapy that improves aging physiology may still produce tissue-specific or compensatory expression patterns that do not mirror the original aging mark.

Translational and clinical implications

Objective 4

Translational interpretation was limited to candidate prioritization, experimental validation, and future research directions; the current data were not used to infer clinical efficacy.

The translational value of CPT2 lies in its specificity. Many aging interventions are discussed at the level of broad pathways: mitochondrial dysfunction, epigenetic drift, inflammation, oxidative stress, or NAD+ decline. These categories are useful but hard to validate clinically because they include many genes and mechanisms. CPT2 offers a narrower candidate. It has a defined mitochondrial role, a measurable transcript and protein, a functional assay through fatty-acid oxidation flux, and a chromatin hypothesis that can be tested by ChIP-qPCR or CUT&Tag.

This is relevant to aging brain research because neurons and glia depend on metabolic flexibility, even if their substrate preferences differ across cell types and states. Bioenergetic limitation is also relevant to cognitive aging and neurodegenerative vulnerability. NAD+ biology has been discussed in relation to metabolism and neurodegeneration, but the field still needs markers that connect intervention response to specific cellular mechanisms [5]. CPT2 could serve as one such marker if its regulation is validated in the appropriate cells and tissues.

The psychiatric relevance should be stated carefully. Any relevance to treatment-resistant depression, metabolic-psychiatric comorbidity, or broader neuropsychiatric conditions is speculative and represents a future research direction only. The current datasets contain no direct transcriptomic, epigenetic, behavioral, or clinical data linking CPT2 or the concordant gene set to psychiatric outcomes. Treatment-resistant depression and metabolic-psychiatric comorbidity often raise questions about mitochondrial stress, inflammatory tone, and impaired adaptive plasticity, but this analysis did not test depression, behavior, or psychiatric phenotypes. If CPT2 rescue is confirmed in neuronal or stress-relevant models, it could justify future disease-relevant experiments; it does not currently support a psychiatric treatment claim.

CPT2 also has biomarker potential. A translational biomarker could include CPT2 mRNA, CPT2 protein, fatty-acid oxidation capacity, or H3K27me3 status at CPT2-linked regulatory regions. A stronger biomarker panel would combine CPT2 with secondary concordant genes such as ACD, ZFAND2B, FMO2, SYNGR2, DUSP11, APBA2, MAP2K2, SPG21, HS3ST6, and PKNOX1. However, because the concordant set had zero significant enrichment terms, it should be treated as a candidate panel rather than a pathway signature.

The clinical advantage of this approach is restraint. Instead of claiming that NMN is broadly anti-aging or that it reverses neuronal epigenetic aging, the analysis identifies one high-priority node with a clear validation path. That is more useful for translational work. It allows future experiments to ask specific questions: Does CPT2 expression decline or become constrained with age? Does NMN restore CPT2 expression in old tissues? Does H3K27me3 at CPT2-linked regions change with age and treatment? Does CPT2 modulation alter fatty-acid oxidation or cellular stress resilience? These are tractable questions.

Limitations and future directions

The most important limitation is the size of the aging target set. With 21,155 target genes, the H3K27me3 program is too broad for simple binary overlap testing. Future work should use a more selective aging target set, such as the top 500, 1,000, or 2,000 genes ranked by ABC score multiplied by absolute K27me3 log-fold change. GAIN and LOSS genes should be tested separately, and promoter-proximal H3K27me3 should be evaluated separately from distal ABC-style assignments.

The second limitation is the NMN signal. No tissue-specific differential result reached FDR < 0.05 for age, NMN at 6 months, or interaction. The rescue genes were therefore based on relaxed nominal criteria. The robust two-tissue filter helps, but it does not replace independent replication. Future NMN analyses should use larger sample sizes, RNA-seq where available, and models that can estimate tissue-specific and shared treatment effects more reliably.

The third limitation is cross-tissue inference. Neuronal chromatin aging cannot be assumed to map directly onto liver, muscle, or white adipose tissue expression. The present analysis should therefore be followed by direct testing in matched tissues or cell systems. For CPT2, the immediate validation steps are qPCR and Western blot in independent aging and NMN cohorts, K27me3 ChIP-qPCR or CUT&Tag-qPCR at implicated CPT2 regulatory regions, and fatty-acid oxidation assays using Seahorse flux or labeled palmitate. Causal tests should include CPT2 knockdown or overexpression with and without NMN. Secondary validation can use a targeted panel of the top concordant genes.

The ABC-style mapping introduces an additional source of uncertainty. The ABC model was developed primarily as a predictive enhancer-promoter framework [18], whereas the present pipeline used an ABC-style score to assign age-associated H3K27me3 regions, a repressive mark, to genes. Distal predictions can be false-positive, incomplete, or low-confidence, particularly when chromatin contact, cell-type specificity, or the regulatory effect of the mark is not directly measured. A predicted region-gene link should therefore not be treated as proof of direct regulation.

This uncertainty could contribute to directional discordance between the neuronal H3K27me3 dataset and the peripheral-tissue expression dataset. Some discordant calls may reflect tissue biology, indirect NMN responses, or compensatory regulation, but some may also be partly technical or artifactual consequences of low-confidence distal assignment. Locus-specific chromatin and expression assays in matched neuronal and peripheral models are needed to separate these possibilities.

A final statistical improvement is to recompute overlap using a valid universe. The appropriate universe should include genes measured in the GSE85718 expression matrix and eligible for GSE190102 region-gene mapping. Permutation-based overlap and rank-based enrichment would be preferable to a simple hypergeometric test. Until those refinements are complete, all conclusions should remain hypothesis-generating.

## Conclusions

This integrative analysis found limited global convergence between age-associated neuronal H3K27me3 remodeling and cross-tissue NMN-rescue transcriptional changes. Although 23 genes were shared, the aging H3K27me3 target set was extremely broad, the initial hypergeometric universe was invalid, and no shared pathway enrichment was detected. These results do not support a claim that NMN broadly reverses neuronal epigenetic aging. The main value of the analysis is candidate discovery. Directional interpretation under repressive H3K27me3 logic identified 14 concordant genes. Among them, CPT2 stood out as the strongest candidate because it combined age-associated H3K27me3 gain, NMN-induced expression increase in old animals, mitochondrial fatty-acid oxidation biology, and the highest downstream validation priority score. The mitochondrial enrichment signal was only nominal, so CPT2 should be described as a focused mechanistic lead rather than proof of a broad mitochondrial program.

The next step is direct validation. CPT2 expression, protein abundance, fatty-acid oxidation function, and H3K27me3 status at CPT2-linked regions should be tested in independent aging and NMN models. If confirmed, CPT2 may provide a practical bridge between epigenetic aging and metabolic rescue, with potential relevance to aging-related bioenergetic decline and future translational work in metabolic conditions. Any relevance to neuropsychiatric conditions remains speculative and requires direct disease-relevant testing; the present datasets do not establish such a link.

## Appendices

Appendix 1: master analysis overview

**Table 6:** Master analysis overview. H3K27me3 is treated as a repressive mark. Therefore, K27me3 gain with age is interpreted as increased repression, whereas K27me3 loss with age is interpreted as de-repression.

Item	Result
Generated	2026-06-30 16:50:59
Workspace	/content/AGING_vs_NMN_MASTER
NMN stage	Completed
Aging stage	Completed
Aging dataset	GSE190102; neuronal H3K27me3 / K27me3 aging signal; 24M versus 3M contrast; ABC-style region–gene mapping.
NMN dataset	GSE85718; long-term oral NMN, 300 mg/kg/day; C57BL/6N mice; skeletal muscle, liver, and white adipose tissue; 6M and 12M; model expression approximately age × treatment.
Main interpretation	Limited global convergence, no shared significant pathway terms, but a small shared set of 23 genes was identified. CPT2 emerged as the strongest validation candidate.

Appendix 2: key findings and principal statistical constraints

**Table 7:** Key findings from the master and downstream summaries.

Analysis component	Value	Interpretation
Aging H3K27me3 target genes	21,155	Very broad target set; nearly genome-wide, limiting binary overlap interpretation.
NMN robust rescue genes	35	Genes showing sign-reversal rescue in at least two tissues using relaxed nominal criteria.
Shared genes	23	Genes present in both the aging K27me3 target set and the robust NMN-rescue set.
Jaccard index	0.0011	Very small exact-gene overlap relative to union size.
Hypergeometric p-value	NaN	Invalid under the stated universe because the universe was 20,000 while the aging target set contained 21,155 genes.
Aging significant enrichment terms	489	Aging target set was broadly pathway-enriched.
NMN significant enrichment terms	0	No significant terms detected for the 35 robust NMN-rescue genes.
Shared significant enrichment terms	0	No pathway-level convergence detected under the applied thresholds.
Direction concordance	14 concordant; 9 discordant; 0 undetermined	Directional interpretation was based on repressive H3K27me3 logic.
Mitochondrial core overlap, master analysis	6 genes	CPT2, CS, NDUFV1, POLG, SUCLA2, and TFB2M overlapped between aging mitochondrial targets and NMN mitochondrial annotation.
Mitochondrial core overlap, strict downstream shared set	1 gene; p = 7.32 × 10^-2^	CPT2 was the only mitochondrial-core gene among the 23 shared downstream genes; enrichment was nominal, not conventionally significant.
Top validation candidate	CPT2	Concordant, mitochondrial, K27me3 gain, NMN-induced expression increase in old animals, and highest downstream priority score.

Appendix 3: NMN-rescue transcriptomic program

**Table 8:** NMN-rescue transcriptomic program. Probe-to-gene processing yielded 18,120 genes from 25,697 probes. Values were treated as already compressed; log2 transformation was skipped and quantile normalization was applied.

Tissue	Age	Treatment	n	Differential genes at FDR < 0.05
Liver	6M	Control	4	Age effect: 0; NMN at 6M: 0; age × treatment interaction: 0.
Liver	6M	NMN	4	
Liver	12M	Control	4	
Liver	12M	NMN	4	
Skeletal muscle	6M	Control	4	Age effect: 0; NMN at 6M: 0; age × treatment interaction: 0.
Skeletal muscle	6M	NMN	4	
Skeletal muscle	12M	Control	4	
Skeletal muscle	12M	NMN	4	
White adipose tissue	6M	Control	4	Age effect: 0; NMN at 6M: 0; age × treatment interaction: 0.
White adipose tissue	6M	NMN	4	
White adipose tissue	12M	Control	4	
White adipose tissue	12M	NMN	4	

Appendix 4: NMN-rescue genes by tissue and robust filtering

**Table 9:** NMN-rescue genes by tissue and robust cross-tissue filtering.

Tissue or category	Number of genes	Criterion or note
Skeletal muscle	421	Age-associated in controls plus sign-reversal interaction; relaxed nominal p criterion.
Liver	355	Age-associated in controls plus sign-reversal interaction; relaxed nominal p criterion.
White adipose tissue	397	Age-associated in controls plus sign-reversal interaction; relaxed nominal p criterion.
Robust rescue genes	35	Present in at least two tissues.
Rescued in all three tissues	0	No robust gene was rescued across all three tissues.
NMN mitochondrial annotation	6	CPT2, CS, NDUFV1, POLG, SUCLA2, TFB2M.

Appendix 5: top robust NMN-rescue genes

**Table 10:** Top robust NMN-rescue genes by absolute mean interaction coefficient.

Gene	Tissues	Mean interaction coefficient	Mean age-control coefficient
MYO15	2	+6.040	-3.606
CMA2	2	+5.783	-3.744
SLC13A5	2	+5.049	-3.654
0610012A05RIK	2	+4.473	-4.378
WAP	2	-4.394	+3.772
APBA2	2	+4.248	-3.594
D430042O09RIK	2	+3.879	-4.566
OLFR627	2	+3.878	-3.787
1700024D23RIK	2	+3.821	-3.820
RAB11A	2	+2.241	-2.286
LOC192950	2	+1.804	-1.754
V1RE1	2	+1.161	+0.114
OLFR508	2	-0.878	-0.064
HS3ST6	2	+0.398	+0.145
6330581L23RIK	2	+0.317	-0.341
CPT2	2	+0.201	-0.141
SYNGR2	2	-0.180	+0.254
TRP53INP2	2	+0.145	-0.209
MOBKL2C	2	-0.134	+0.236
2810405F18RIK	2	+0.123	-0.095
2810441K11RIK	2	-0.118	+0.122
PKNOX1	2	-0.110	+0.263
SPG21	2	-0.110	+0.128
PSME3	2	+0.100	-0.221
0610009O03RIK	2	-0.096	+0.233

Appendix 6: aging H3K27me3 parameters

**Table 11:** Aging H3K27me3 epigenetic program.

Parameter	Value	Note
Histone mark	K27me3 / H3K27me3	Repressive mark; not treated as active enhancer signal.
Samples per age	3M: 5; 12M: 5; 24M: 5	Age groups parsed from 15 K27me3 bigWig files.
Consensus regions	366,721	Peak-union regions used for quantification.
Differential mode	Relaxed p < 0.01	24M versus 3M contrast.
Age-associated regions	9,752	Regions selected for age-associated K27me3 change.
Progressive loss clusters	1, 4	K27me3 signal progressively decreased with age.
Progressive gain clusters	0, 3	K27me3 signal progressively increased with age.
Regions carried to ABC	6,402	Progressive gain or loss regions used for region–gene scoring.
Protein-coding genes	21,674	GENCODE vM25 protein-coding genes considered.
ABC region–gene pairs	427,099	Predictions above the ABC-style score threshold.
Aging K27me3 target genes	21,155	GAIN = 9,178; LOSS = 12,470.

Appendix 7: mitochondrial-core genes

**Table 12:** Mitochondrial-core genes in the aging target set.

Count	Genes
66	ACADL, ACADM, ACADVL, ACO2, ATP5A1, ATP5B, ATP5C1, ATP5J, ATP5O, COX4I1, COX5A, COX5B, COX6A1, COX7A1, COX8B, CPT1B, CPT2, CS, CYC1, CYCS, DLAT, DNM1L, ECI1, ESRRA, FH1, HADHA, HADHB, IDH3A, MDH2, MFN1, MFN2, MRPL12, MRPS12, NDUFA1, NDUFA2, NDUFA9, NDUFB8, NDUFS1, NDUFS3, NDUFV1, NRF1, OGDH, OPA1, PDHA1, PDHB, PINK1, POLG, PPARGC1A, PRKN, SDHA, SDHB, SLC25A20, SLC25A4, SOD2, SUCLA2, TFAM, TFB1M, TFB2M, TIMM23, TOMM20, UCP2, UCP3, UQCRC1, UQCRC2, VDAC1, VDAC2.

Appendix 8: gene-set overlap

**Table 13:** Gene-set overlap between aging H3K27me3 targets and robust NMN-rescue genes.

Metric	Value	Comment
Aging K27me3 targets	21,155	Very broad target set.
NMN-rescue genes	35	Robust genes present in at least two tissues.
Intersection	23	Shared genes carried into downstream analysis.
Jaccard index	0.0011	Very small overlap relative to total union.
Hypergeometric p-value	NaN	Formal test invalid because the stated universe was smaller than the aging target set.

Appendix 9: shared-gene direction and concordance

**Table 14:** Shared genes with direction and concordance classification. Concordance was defined as GAIN plus positive NMN interaction, or LOSS plus negative NMN interaction. This follows the interpretation that H3K27me3 gain increases repression and H3K27me3 loss decreases repression.

Gene	Aging K27me3 direction	NMN effect in old animals	NMN sign	Verdict
ACD	LOSS	NMN lowers old	-1.0	Concordant
APBA2	GAIN	NMN raises old	+1.0	Concordant
BCHE	GAIN	NMN raises old	+1.0	Concordant
CMA2	LOSS	NMN raises old	+1.0	Discordant
CPT2	GAIN	NMN raises old	+1.0	Concordant
DUSP11	LOSS	NMN lowers old	-1.0	Concordant
FMO2	LOSS	NMN lowers old	-1.0	Concordant
GALR1	GAIN	NMN raises old	+1.0	Concordant
HS3ST6	GAIN	NMN raises old	+1.0	Concordant
MAP2K2	LOSS	NMN lowers old	-1.0	Concordant
MYO15	LOSS	NMN raises old	+1.0	Discordant
MYOM2	LOSS	NMN lowers old	-1.0	Concordant
OLFR508	GAIN	NMN lowers old	-1.0	Discordant
PKNOX1	LOSS	NMN lowers old	-1.0	Concordant
PSME3	LOSS	NMN raises old	+1.0	Discordant
RAB11A	LOSS	NMN raises old	+1.0	Discordant
RANGAP1	LOSS	NMN raises old	+1.0	Discordant
SLC13A5	LOSS	NMN raises old	+1.0	Discordant
SPG21	LOSS	NMN lowers old	-1.0	Concordant
SYNGR2	LOSS	NMN lowers old	-1.0	Concordant
TRP53INP2	LOSS	NMN raises old	+1.0	Discordant
WAP	GAIN	NMN lowers old	-1.0	Discordant
ZFAND2B	LOSS	NMN lowers old	-1.0	Concordant

Appendix 10: pathway and enrichment comparison

**Table 15:** Pathway and enrichment comparison.

Gene set	Significant terms	Interpretation
Aging H3K27me3 targets	489	Broad enrichment across transport, signaling, mitochondrial/energy, immune, synaptic, developmental, and other biological themes.
Robust NMN-rescue genes	0	No significant enrichment terms detected, likely influenced by small set size.
Shared significant terms	0	No pathway-level convergence detected.
Shared genes only	0	No significant terms in GO, KEGG, Reactome, Hallmark, or MGI libraries.
Concordant genes	0	No significant enrichment among the 14 concordant genes.
Discordant genes	38	Significant GO terms were detected, mainly involving transport, endosomal trafficking, receptor transport, and microtubule-related processes.

Appendix 11: top aging enrichment terms

**Table 16:** Top aging enriched terms.

Library	Term	Adjusted p-value
KEGG	ABC transporters	2.35 × 10^-37^
Reactome	Ion Channel Transport R-HSA-983712	3.98 × 10^-32^
KEGG	Adrenergic signaling in cardiomyocytes	2.29 × 10^-27^
Reactome	Ion Transport By P-type ATPases R-HSA-936837	2.53 × 10^-25^
Reactome	Transport Of Small Molecules R-HSA-382551	1.74 × 10^-24^
KEGG	Aldosterone synthesis and secretion	6.53 × 10^-19^
Reactome	RHOA GTPase Cycle R-HSA-8980692	8.11 × 10^-19^
GO	Adenylate cyclase-activating G protein-coupled receptor signaling pathway	1.34 × 10^-16^
Reactome	Insulin Receptor Recycling R-HSA-77387	2.18 × 10^-16^
KEGG	Collecting duct acid secretion	3.08 × 10^-16^
KEGG	Salivary secretion	1.64 × 10^-15^
Reactome	Defective B3GALTL Causes PpS R-HSA-5083635	9.82 × 10^-15^
GO	Regulation Of Small GTPase Mediated Signal Transduction	1.45 × 10^-14^
KEGG	cGMP-PKG signaling pathway	1.72 × 10^-14^
Reactome	O-glycosylation Of TSR Domain-Containing Proteins R-HSA-5173214	2.23 × 10^-14^

Appendix 12: functional theme counts

**Table 17:** Functional theme counts among significant enrichment terms.

Functional theme	Aging	NMN	Shared
Mitochondrial/Energy	11	0	0
Chromatin/Epigenetic	1	0	0
Inflammaging/Immune	5	0	0
Synaptic/Plasticity	15	0	0
Aging/Senescence	1	0	0
Development/Morphogen	4	0	0
Signaling	10	0	0

Appendix 13: downstream validation shortlist

**Table 18:** Downstream validation shortlist. Priority score was defined in the downstream analysis as 3 × concordant + minimum number of tissues, capped at 3 + 2 × ABC rank + 2 × absolute interaction rank + mitochondrial membership.

Gene	Priority	Verdict	Tissues	Mean NMN interaction	Age-control coefficient	ABC score
CPT2	7.91	Concordant	2	+0.201	-0.141	6.98 × 10^-2^
ACD	7.35	Concordant	2	-0.082	+0.122	1.77 × 10^-1^
ZFAND2B	7.35	Concordant	2	-0.065	+0.138	1.88 × 10^-1^
FMO2	7.26	Concordant	2	-0.020	+0.023	2.64 × 10^-1^
SYNGR2	7.17	Concordant	2	-0.180	+0.254	8.45 × 10^-2^
DUSP11	6.91	Concordant	2	-0.084	+0.105	9.28 × 10^-2^
APBA2	6.83	Concordant	2	+4.248	-3.594	4.01 × 10^-2^
MAP2K2	6.83	Concordant	2	-0.088	+0.179	8.80 × 10^-2^
SPG21	6.74	Concordant	2	-0.110	+0.128	8.37 × 10^-2^
HS3ST6	6.65	Concordant	2	+0.398	+0.145	4.57 × 10^-2^
PKNOX1	6.57	Concordant	2	-0.110	+0.263	5.91 × 10^-2^
MYOM2	6.57	Concordant	2	-0.011	-0.741	1.02 × 10^-1^
GALR1	5.78	Concordant	2	+0.062	-0.673	5.17 × 10^-2^
BCHE	5.70	Concordant	2	+0.084	+0.009	3.69 × 10^-2^
SLC13A5	5.57	Discordant	2	+5.049	-3.654	1.74 × 10^-1^
CMA2	5.48	Discordant	2	+5.783	-3.744	1.18 × 10^-1^
WAP	4.87	Discordant	2	-4.394	+3.772	9.05 × 10^-2^
TRP53INP2	4.61	Discordant	2	+0.145	-0.209	1.16 × 10^-1^
PSME3	4.52	Discordant	2	+0.100	-0.221	1.20 × 10^-1^
OLFR508	4.35	Discordant	2	-0.878	-0.064	8.39 × 10^-2^
MYO15	4.35	Discordant	2	+6.040	-3.606	4.77 × 10^-2^
RAB11A	4.26	Discordant	2	+2.241	-2.286	7.83 × 10^-2^
RANGAP1	3.39	Discordant	2	+0.003	-0.012	1.01 × 10^-1^

Appendix 14: CPT2-focused result summary

**Table 19:** CPT2-focused result summary.

Metric	CPT2 value	Interpretation
Shared gene status	Shared	Present in both the aging K27me3 target set and the robust NMN-rescue set.
Aging K27me3 direction	GAIN	Consistent with increased age-associated repressive chromatin signal.
K27me3 LFC, 24M versus 3M	+3.504	Large positive age-associated K27me3 change.
NMN effect	NMN raises old	Positive expression interaction in old animals, consistent with rescue under repressive-mark logic.
Mean NMN interaction coefficient	+0.201	Expression increased with NMN in old animals.
Mean age-control coefficient	-0.141	Age effect in control animals was negative on average.
Number of NMN tissues	2	Met robust criterion of presence in at least two tissues.
ABC score	0.0698	Region–gene support from the downstream aging target table.
Mitochondrial-core status	Yes	Only mitochondrial-core gene in the strict 23-gene shared downstream set.
Downstream priority score	7.91	Highest-ranked validation candidate.

Appendix 15: discordant enrichment terms

**Table 20:** Discordant enrichment terms. The downstream summary reported 38 significant discordant-set terms. The table lists the terms explicitly shown in the provided summary.

Library	Term	Adjusted p-value
GO	Succinate Transport	3.70 × 10^-2^
GO	Response To Lithium Ion	3.70 × 10^-2^
GO	Neurotransmitter Receptor Transport, Endosome To Postsynaptic Membrane	3.70 × 10^-2^
GO	Neurotransmitter Receptor Transport, Endosome To Plasma Membrane	3.70 × 10^-2^
GO	Plasma Membrane To Endosome Transport	3.70 × 10^-2^
GO	Astral Microtubule Organization	3.70 × 10^-2^
GO	Neurotransmitter Receptor Transport To Postsynaptic Membrane	3.70 × 10^-2^
GO	Regulation Of Endosome Size	3.70 × 10^-2^
GO	C4-dicarboxylate Transport	3.70 × 10^-2^
GO	Negative Regulation Of Nucleocytoplasmic Transport	3.70 × 10^-2^
